# Consistent effects of independent domestication events on the plant microbiota

**DOI:** 10.1016/j.cub.2023.12.056

**Published:** 2024-01-16

**Authors:** Riccardo Soldan, Marco Fusi, Massimiliano Cardinale, Felix Homma, Luis Guillermo Santos, Peter Wenzl, Marcel Bach-Pages, Elena Bitocchi, Maria Isabel Chacon Sanchez, Daniele Daffonchio, Gail M. Preston

**Affiliations:** 1 University of Oxford, Department of Biology, Oxford, UK; 2 Center for Conservation and Restoration Science, Edinburgh Napier University, Edinburgh, UK; 3 University of Salento, Department of Biological and Environmental Sciences and Technologies, Lecce, Italy; 4 The Alliance Biodiversity International and the International Center for Tropical Agriculture (CIAT), Palmira, Colombia; 5 Dipartimento di Scienze Agrarie, Alimentari ed Ambientali, Università Politecnica delle Marche, Ancona, Italy; 6 Departamento de Agronomía, Facultad de Ciencias Agrarias, Universidad Nacional de Colombia, Bogotá, Colombia; 7 Red Sea Research Center (RSRC), 4700 King Abdullah University of Science and Technology (KAUST), Thuwal 23955-6900, Saudi Arabia

## Abstract

The effect of plant domestication on plant-microbe interactions remains difficult to prove. In this study, we provide evidence of a domestication effect on the composition and abundance of the plant microbiota. We focused on the genus *Phaseolus*, which underwent four independent domestication events within two species (*P. vulgaris* and *P. lunatus*), providing multiple replicates of a process spanning thousands of years. We targeted *Phaseolus* seeds to identify a link between domesticated traits and bacterial community composition as *Phaseolus* seeds have been subject to large and consistent phenotypic changes during these independent domestication events. The seed bacterial communities of representative plant accessions from subpopulations descended from each domestication event were analyzed under controlled and field conditions. The results showed that independent domestication events led to similar seed bacterial community signatures in independently domesticated plant populations, which could be partially explained by selection for common domesticated plant phenotypes. Our results therefore provide evidence of a consistent effect of plant domestication on seed microbial community composition and abundance and offer avenues for applying knowledge of the impact of plant domestication on the plant microbiota to improve microbial applications in agriculture.

## Introduction

Plant microbiomes can extend host evolutionary potential,^1^ playing pivotal roles in plant growth and stress tolerance.^2^ Evidence shows that plant microbiomes can enhance drought resilience^3^ and disease resistance,^4–6^ making them essential for sustainable agricultural production.^7^ Virtually, all vegetables, grains, and fruits we rely upon come from domesticated plants, which genetically and often phenotypically differ from their wild counterparts.^8^ For example, changes in fruit size, reproductive strategy, flowering time, mineral contents, secondary metabolites, and seed-shattering are often observed in domesticated plants.^8^ Because the effect of plant genes extends beyond the individual plant and influences microbial communities,^9^ it is logical to hypothesize that changes in plant traits selected during domestication could also drive changes in microbiota composition.^10^ Furthermore, as domesticators are likely to select for common traits, such as increased seed size, palatability, or early maturity, it is also logical to hypothesize that independent domestication events for the same plant species, which are focused on the same plant product (e.g., seeds or fruits), could lead to similar changes in the plant microbiota, with potential consequences for plant health and productivity.^10^ Ultimately, a better understanding of domesticated plant microbiomes can foster microbial applications in agriculture^11^ through, for example, the development of more effective microbial bioinoculants.

Recent studies have proved a plant domestication effect on plant microbiota composition^12–15^ and have also provided evidence that these changes between wild and domesticated plant microbial communities could be linked to differences in plant traits, such as root length^16^ and plant height.^17^ However, drawing general conclusions would require studying the microbiota of plants that have been domesticated independently, ideally several times. This is challenging as domestication is a process lasting thousands of years.

Among different domesticated plants, the genus *Phaseolus* has a well-defined population structure, and two of its species, namely *Phaseolus vulgaris* (common bean) and *P. lunatus* (Lima bean), have been domesticated at least twice independently,^18,19^ over two geographical areas in the Americas. As a result, for each plant species, two gene pools exist (Mesoamerican and Andean gene pools), containing both wild and domesticated accessions. This distinctive feature of four independent domestication events that have occurred within two congeneric species offers the possibility of statistically testing the effect of domestication on plant microbiota.

To investigate the plant traits associated with members of the microbial community, one approach is to treat them as quantitative trait loci (QTL).^2^ Although this approach is paving the way to disentangling the genetic basis of microbiota assembly, it remains technically challenging on a broad scale (hundreds of different plant accessions, which require vast genotyping and phenotyping efforts). Amplicon sequencing using marker genes (e.g., 16S rRNA) to determine the effect of domestication on microbiota composition and statistical modeling to disentangle drivers represents an essential precursor.

For these reasons, we focused on amplicon sequencing of the *Phaseolus* bacteriota and selected accessions based on the population genetic structure of both wild and domesticated plants,^18,20–23^ thus ensuring a good representation of the known genetic diversity within *P. vulgaris* and *P. lunatus*.

We selected the seed as the plant compartment for analysis based on our previously described evolutionary framework,^10^ which contextualizes host control of the microbiome for domesticated plants as a ‘double-leash’ acting from domesticator to host and host to microbes. Essentially, our framework, which expands the framework of Foster et al.,^24^ predicts that microbiota assembly in domesticated crops could be highly influenced by domesticated plant phenotypes. In *Phaseolus* spp. seeds, multiple traits, including seed size,^25,26^ chemical,^27,28^ and mineral composition^29,30^ have been targeted by domesticators, leading to remarkable phenotypic differences.

We investigated whether parallel domestication events, selecting for quantitively measurable seed traits had consistent effects on seed bacterial community composition and abundance, an important component of the plant microbiota. We first studied seed bacterial communities for wild and domesticated plants grown under controlled environmental conditions and then examined whether these differences observed under controlled conditions could also be found in seeds from a wider range of plant genotypes grown in the field (procured directly from the CGIAR-CIAT germplasm bank in Colombia). Our results suggest that domestication consistently influenced plant microbiota composition and abundance across multiple domestication events and within plant species. The differences in wild and domesticated bacterial communities appear to be partially explained by the selection of common plant traits during domestication.

## Results

### Independent domestication events consistently influence seed bacterial community composition in greenhouse-grown *P. vulgaris*

**Figure 1:**
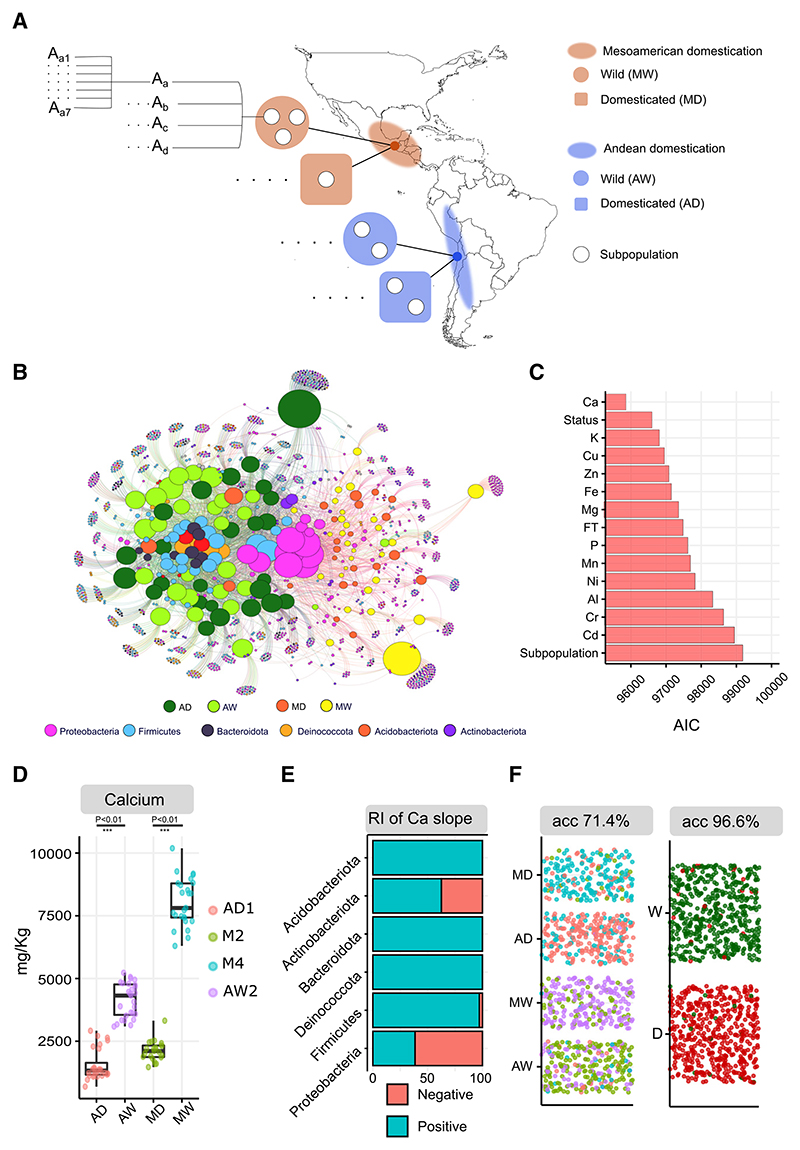
Parallel domestication events consistently influence seed bacterial community composition through domesticated plant phenotypes in greenhouse-grown *P. vulgaris* (A) Two gene pools of *Phaseolus vulgaris* exist. One in the Andes and one in Mesoamerica. Each gene pool has undergone an independent domestication event. We selected one subpopulation per biological status (domesticated and wild) and domestication event (Andes and Mesoamerica) and selected four accessions per subpopulation (A_a_, A_b_, A_c_, and A_d_) with seven biological replicates each (A_a1_, A_a2_, …) and grew them in the same soil under greenhouse conditions. Seeds were collected at maturity and extracted directly from the pod under axenic conditions for seed microbiota analysis. (B) Bipartite network representing sample/sequence variant interactions. In the network, node size is proportional to the number of degrees (number of connections). In the network, central nodes indicate the core microbiome, which in this case is mainly made of Proteobacteria, in particular *Pseudomonadaceae* (Figure S1A). AD, Andean domesticated; AW, Andean wild; MD, Mesoamerican domesticated; MW, Mesoamerican wild. (C) Sum of the AIC values of the multiple generalized linear models (GLMs) used to explain the seed microbiota composition. The explanatory variable used in each model is represented on the y axis. For all quantitative explanatory variables (e.g., mineral concentration and flowering time [FT]), two parameters are being estimated, namely, intercept and slope. Explanatory variable biological status (status) has two levels (wild and domesticated), whereas the explanatory variable subpopulation has 4 levels (AD1, AW1, MW4, and M2). The multivariate property of the data is accounted for in the calculation of the p value by the resampling procedure. (D) Concentration of calcium (Ca) in plant seeds. AD, Andean domesticated; AW, Andean wild; MD, Mesoamerican domesticated; MW, Mesoamerican wild. The nomenclature of subpopulation names (e.g., AD1 and M4) follow the nomenclature used in Rodriguez et al.^22^ Welch’s t test was used to assess the statistical significance of differences between means. Standard deviations in mg kg^−1^ are 608, 686, 402, and 963 for AD, AW, MD, and MW, respectively. (E) Percentage of microbial taxa (0%–100%) that were negatively or positively affected by Ca concentration after filtering sequence variants (SVs) based on 5% prevalence. RI, relative importance. (F) Accuracy and confusion matrix of the random forest classifier (10 times 5-fold cross-validation) for classification task domestication status (2 levels; W, wild; D, domesticated) and domestication status within domestication event (DS|DE) (4 levels; AD, Andean domesticated; AW, Andean wild; MD, Mesoamerican domesticated, MW, Mesoamerican wild, which, in this case, also correspond to subpopulation). See also Data S1A and Table S1 and Figures S1 and S3.

Because both environmental and genetic factors are known to influence the composition of the seed microbiota, in our first experiment, we aimed to compare the effect of independent domestication events on seed bacterial community composition for wild and domesticated plants grown under controlled conditions. We selected four representative subpopulations,^22^ one for each gene pool (Andean and Mesoamerican) and biological status (wild or domesticated) of *P. vulgaris*, namely, AD1 (subpopulation belonging to the domesticated Andean gene pool, AD), AW1 (subpopulation belonging to the wild Andean gene pool, AW), M2 (subpopulation belonging to the domesticated Mesoamerican gene pool, MD), MW4 (subpopulation belonging to the wild Mesoamerican gene pool, MW), and four different accessions per subpopulation with seven biological replicates (112 total plants) and grew them under controlled greenhouse conditions (Figure 1A). The selection of the accessions was based on previous studies on the population genetic structure of *P. vulgaris*^22^ to avoid genotypically admixed accessions (Data S1).

Amplicon sequencing results of the V4 hypervariable region of the 16S rRNA gene yielded high sequencing depth libraries (>5,000 reads per sample). At the same time, to link microbiota composition to plant traits, we measured several plant phenotypes, such as flowering time and seed mineral concentration as a proxy for domesticated plant phenotypes.^31^ Previous studies have already reported a domestication effect on seed cation concentrations,^30,32^ and it is a trait that is consistently measurable.

The seed microbiota of *P. vulgaris* was characterized by high prevalence and dominance of Proteobacteria (Figure 1B), in particular, *Pseudomonadaceae* (Figure S1A), in agreement with previous studies.^33^ To test whether plant domestication influenced seed microbial community composition and abundance, we applied two independent and different approaches, namely, model-based multivariate statistics and machine learning.

Using the multivariate statistic approach, we found that the model that considered subpopulation as an explanatory variable had the worst performance, as indicated by the highest Akaike information criterion (AIC) score (Figure 1C). This result suggests that estimating parameters for each subpopulation (AD-AW-MD-MW) is statistically futile because the seed bacterial community composition is similar between plants with the same biological status (wild or domesticated) regardless of differences in gene pools (e.g., between AD and MD) as indicated by the lower AIC of this model.

When linking plant phenotypes to microbiota composition, we found that the best model based on AIC was the one considering seed calcium (Ca) concentration (likelihood-ratio test = 6,916, p = 0.001***) (Figure 1C). We found Ca concentration in domesticated seeds to be statistically lower than in wild-type seeds (Figure 1D) for both domestication events, supporting the use of Ca concentration as a proxy for genetically determined traits that have changed during domestication. Plant domestication has reduced phenotypic diversity (Figure 1D), leading to both AD1 and M2 subpopulations having similar Ca concentrations compared with their wild counterparts. The model accounting for Ca concentration as an explanatory variable performed better than the one with biological status, suggesting that the differences in seed microbial community composition between MW4 and AW1 subpopulations are stronger than between M2 and AD1.

Most microbial members belonging to the phylum Proteobacteria were statistically negatively influenced by Ca concentration, with lower Ca concentration in domesticated seeds being associated with an increased abundance of Proteobacterial taxa (Figure 1E).

Because model selection based on AIC does not consider correlation across taxa, which is only accounted for in the p value of the test statistic,^34^ we further validated these results by applying Gaussian copula models^35^ to focus on the extent to which explanatory variables (Ca concentration and biological status) would explain co-occurrence patterns (interactions between bacterial community members and environmental factors). Again, we found that the best model was the one accounting for Ca concentration, explaining 31% of the correlations across taxa compared with 26 % explained by biological status (Table S1).

To further validate these results, we used a random forest classifier to predict the biological status (domesticated or wild) and subpopulations (AW2, AD1, M4, and M2) of *P. vulgaris* based on seed microbiota data. The results showed that the classification accuracy was higher for factor domestication status (96.6%) than for factor subpopulation (71.4%) (Figure 1F) in agreement with our multivariate model-based approach. The lowest classification errors were within AD1 and M4 subpopulations (about 23%), which are at the lower and upper boundaries of the phenotypic mean for Ca concentration.

### Independent domestication events consistently influence seed bacterial community composition in field-grown *P. vulgaris*

**Figure 2:**
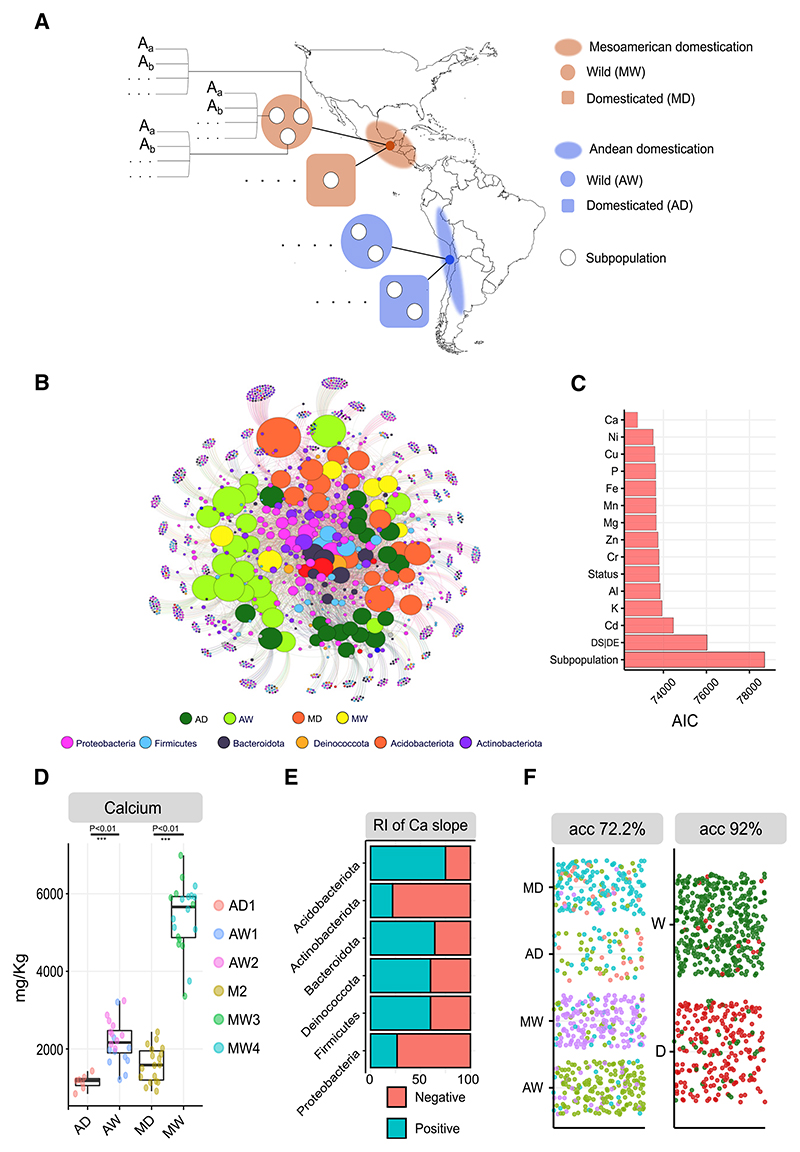
Parallel domestication events consistently influence seed bacterial community composition through domesticated plant phenotypes in field-grown *P. vulgaris* (A) We expanded the previous analysis to select all *P. vulgaris* subpopulations. In this case, we considered as replicates the different plant genotypes (A_a_, A_b_, A, …) within each subpopulation, sampling 70 different genotypes encompassing most of the *P. vulgaris* known genetic diversity. (B) Bipartite network representing sample/sequence variant interactions. In the network, node size is proportional to the number of degrees (number of connections). In the network, central nodes indicate the core microbiome, which in this case is mainly made of Deinococcota, Firmicutes, and Bacteroidota. AD, Andean domesticated; AW, Andean wild; MD, Mesoamerican domesticated; MW, Mesoamerican wild. (C) Sum of the AIC values of the multiple generalized linear models (GLMs) used to explain the seed microbiota composition. The explanatory variable used in each model is represented on the y axis. For all quantitative explanatory variables (e.g., mineral concentration), two parameters are being estimated, namely, intercept and slope. Explanatory variable biological status (status) has two levels (wild and domesticated), whereas the explanatory variable DS|DE (domestication status within domestication event) has four levels (AD, AW, MD, and MW). Explanatory variable subpopulation has six levels. Collection date and regeneration site of the accessions were included as predictors. However, they were not significant (p > 0.05; Table S3); thus, the minimum adequate model included one explanatory variable only. The multivariate property of the data is accounted for in the calculation of the p value by the resampling procedure. (D) Concentration of calcium (Ca) in plant seeds per biological status and domestication event. AD, Andean domesticated; AW, Andean wild; MD, Mesoamerican domesticated; MW, Mesoamerican wild. The nomenclature of subpopulation names (e.g., AD1 and M4) follows the nomenclature used in Rodriguez et al.^22^ Welch’s t test was used to assess the statistical significance of differences between means. Standard deviations in mg kg^−1^ are 162, 551, 436, and 880 for AD, AW, MD, and MW, respectively. (E) Percentage of microbial taxa (0%–100%) that were negatively or positively affected by Ca concentration after filtering SVs based on 5% prevalence. RI, relative importance. (F) Accuracy and confusion matrix of the random forest classifier (10 times 5-fold cross-validation) for classification task domestication status (2 levels; W, wild; D, domesticated) and domestication status within domestication event (4 levels; AD, Andean domesticated; AW, Andean wild; MD, Mesoamerican domesticated; MW, Mesoamerican wild). See also Data S1B and Tables S2 and S3 and Figures S1 and S3.

Because our results showed a clear effect of domestication on the plant microbiota, we investigated whether we could replicate similar results for seeds collected from plants grown in the field (at CGIAR-CIAT, Colombia). We sampled all identified subpopulations of *P. vulgaris*^22^ (AD1, AW1, AW2, MW3, MW1, MW4, and M2; we excluded AD2 because only a few accessions belonged to this subpopulation and were mainly admixed^22^) and considered as replicates different accessions belonging to the same subpopulation (Figure 2A). In total, we analyzed the seed microbiota of 70 different plant genotypes. Seed bacterial communities were characterized by high prevalence of Bacteroidota, Firmicutes, and Deinococcota (Figure 2B) and low abundance of *Pseudomonadaceae* (Figure S1B), in contrast with our previous experiment. The low relative abundance of *Pseudomonadaceae* is likely to be attributed to the seed drying process, which was reported to significantly reduce the abundance of *Pseudomonadaceae* (50% to 0.9% reduction in relative abundance) in seeds,^36^ which largely agrees with our findings.

As in our previous experiment, the best model according to the AIC criteria was the one accounting for Ca concentration (LRT = 3,090, p = 0.0009***), surpassing the model accounting for biological status (wild or domesticated) (Figure 2C). We found once more that plant domestication had significantly reduced seed Ca concentration in both domestication events (Figure 2D), reducing plant phenotypic variation. As per our previous experiment, the majority of the Proteobacterial taxa were negatively affected by increasing Ca concentration (Figure 2E). The Gaussian copula models also confirmed that Ca concentration explained more co-occurrence patterns than biological status, 19%, and 10%, respectively (Table S2).

The random forest classifier further suggests that the composition of microbial communities in domesticated seeds is different from the composition of wild-type seeds, independently of the domestication event (Figure 2F).

The results of both experiments showed that independent domestication processes induce similar bacterial community-level changes, statistically driven by plant traits selected during domestication.

### Independent domestication events consistently influence seed bacterial community composition in field-grown *P. lunatus*

**Figure 3:**
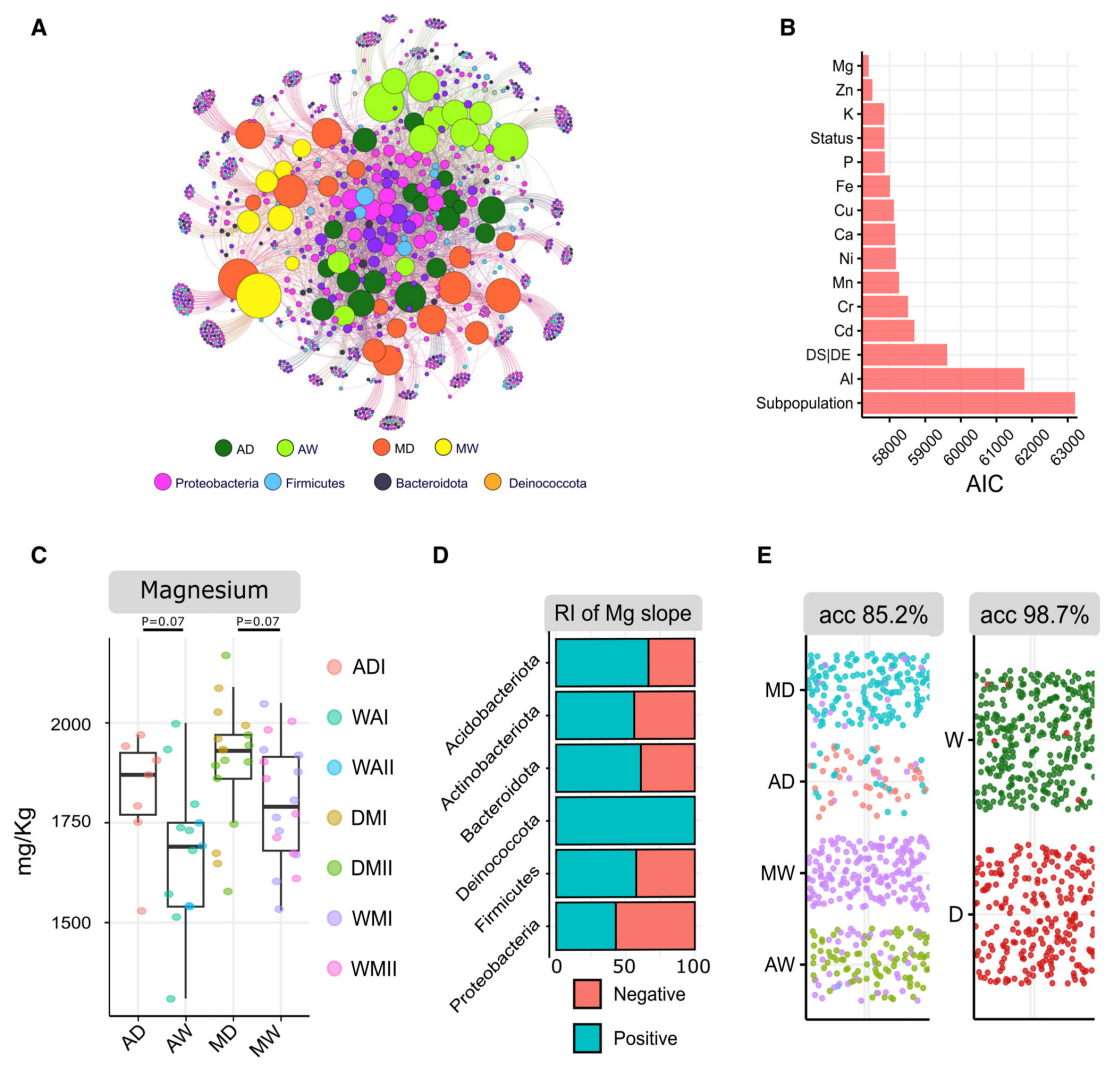
Parallel domestication events consistently influence seed bacterial community composition through domesticated plant phenotypes in field-grown *P. lunatus* (A) Bipartite network representing sample/sequence-variant interactions. In the network, node size is proportional to the number of degrees (number of connections). In the network, central nodes indicate the core microbiome. AD, Andean domesticated; AW, Andean wild;MD, Mesoamerican domesticated; MW, Mesoamerican wild. (B) Sum of the AIC values of the multiple generalized linear models (GLMs) used to explain the seed microbiota composition. The explanatory variable used in each model is represented on the y axis. For all quantitative explanatory variables (e.g., mineral concentration), two parameters are being estimated, namely, intercept and slope. Explanatory variable biological status (status) has two levels (wild and domesticated), whereas the explanatory variable DS|DE (domestication status within domestication event) has four levels (AD, AW, MD, and MW). Predictor subpopulation has six levels. Collection date and regeneration site of the accessions were included as predictors. However, they were not significant (p > 0.05; Table S5), thus the minimum adequate model for all three experiments included one explanatory variable only. (C) Concentration of magnesium (Mg) in plant seeds per biological status and domestication event. AD, Andean domesticated; AW, Andean wild; MD, Mesoamerican domesticated; MW, Mesoamerican wild. The nomenclature of subpopulation names (e.g., ADI and WAII) follow the nomenclature used in Cachó n-Sá nchez and Martínez-Castillo.^23^ Welch’s t test was used to assess the statistical significance of differences between means. Standard deviations in mg kg^−1^ are 151, 184, 155, and 150 for AD, AW, MD, and MW, respectively. (D) Percentage of microbial taxa (0%–100%) that were negatively or positively affected by Mg concentration after filtering SVs based on 5% prevalence. RI, relative importance. (E) Accuracy and confusion matrix of the random forest classifier (10 times 5-fold cross-validation) for classification task domestication status (2 levels; W, wild; D, domesticated) and domestication status within domestication event (4 levels; AD, Andean domesticated; AW, Andean wild; MD, Mesoamerican domesticated; MW, Mesoamerican wild). See also Data S1C and Tables S4 and S5 and Figures S1–S3.

To further test whether these results were species specific, we applied the same conceptual and statistical framework to *P. lunatus* and analyzed the seed microbial communities of plant genotypes belonging to all identified subpopulations for both domestication events (ADI, WAI, WAII, DMI, DMII, WMI, and WMII)^23^ grown at CGIAR-CIAT in Colombia (62 different plant genotypes). Similar to *P. vulgaris, P. lunatus* was also domesticated twice independently in the Andes and Mesoamerica. Seeds of *P. lunatus* appeared to be colonized by taxonomically similar microbes to those found in *P. vulgaris* seeds, but we also detected the presence of *Rhizobiaceae* (Figures 3A and S1C).

*P. lunatus* seed microbiota were statistically influenced by domesticated plant phenotypes and, in particular, by seed magnesium (Mg) concentration (LRT = 2,255, p = 0.001***) (Figure 3B), which was the model with the lowest AIC. Mg concentration was found to be non-statistically higher in domesticated seeds compared with wild seeds for both domestication events (Figure 3C), negatively influencing the majority of Proteobacteria (Figure 3D). The differences in phenotype means between wild and domesticated accessions were less pronounced compared with *P. vulgaris*, leading to only 11% of the co-variation between taxa being explained by Mg concentration (Table S4). Nonetheless, the seeds of *P. lunatus*, similar to those of *P. vulgaris*, have distinctive bacterial signatures that can be used to accurately predict if seeds are wild or domesticated (Figure 3F), indicating a consistent effect of domestication on seed bacterial communities. The results of the random forest classifier were not strongly influenced by a slight imbalance (fewer samples in the AD group) (Figure S2).

### Microbial signatures introduced by plant domestication include members of the Proteobacteria, Firmicutes, and Bacteroidota phyla

**Figure 4:**
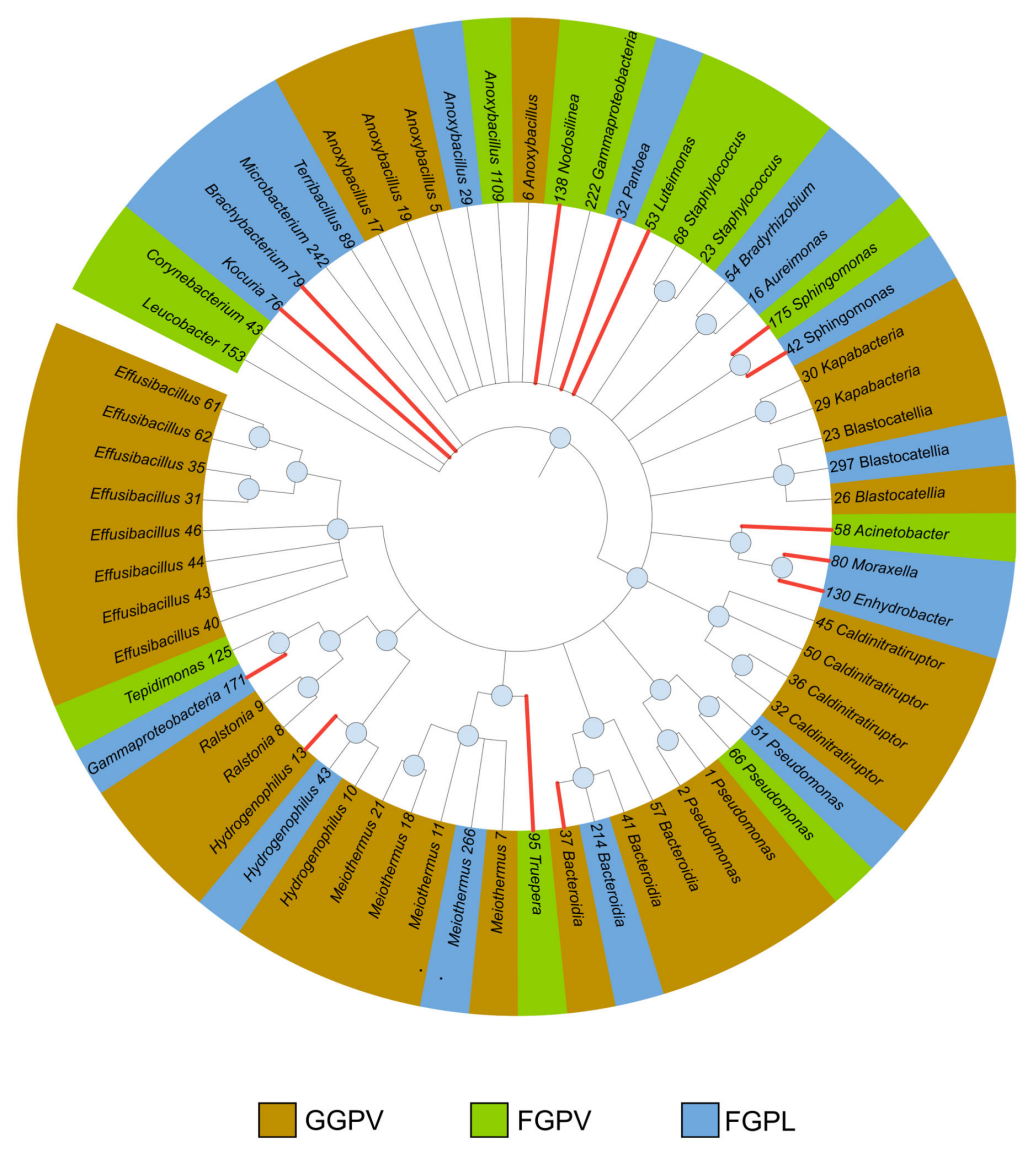
Minimal set of microbial taxa can be used to accurately distinguish wild vs. domesticated plants Maximum likelihood phylogenetic tree showing the indicator taxa selected by the Boruta algorithm for all three experiments. The Boruta algorithm is employed to determine the most critical microbial members that can effectively differentiate between wild and domesticated plants. Red lines indicate the sub-selection of the indicator taxa used for the random forest-based classifier. Branches with bootstrap values lower than 75% are not shown. Internal node sizes are proportional to bootstrapping values (>75%). The microbial features used in the random forest classifier and that were pre-selected by the Boruta algorithm identify the minimal set of microbial members necessary for classifying samples into wild and domesticated plants.

The minimum set of microbial members that allowed high accuracy in the classification of wild vs. domesticated accessions mainly belonged to three phyla, namely, Proteobacteria, Firmicutes, and Bacteroidota. In particular, for the first experiment (greenhouse-grown *P. vulgaris* [GGPV]), two sequence variants (SVs) among the pre-selected features by Boruta alone allowed a classification accuracy of 96.6% (Figure 4). The two SVs, namely, SV_13 and SV_37, belonged to the *Proteobacteria* and *Bacteroidota* phyla, respectively. Furthermore, certain indicator taxa, such as the genus *Pseudomonas* and *Anoxibacillus*, appeared as important features in all three experiments, suggesting a recurrent differential recruitment of these microbial members in the seed microbiome of wild and domesticated plants (Figure 4).

### Changes in bacterial community composition and abundance driven by plant domestication are reflected at a microbial functional level

**Figure 5:**
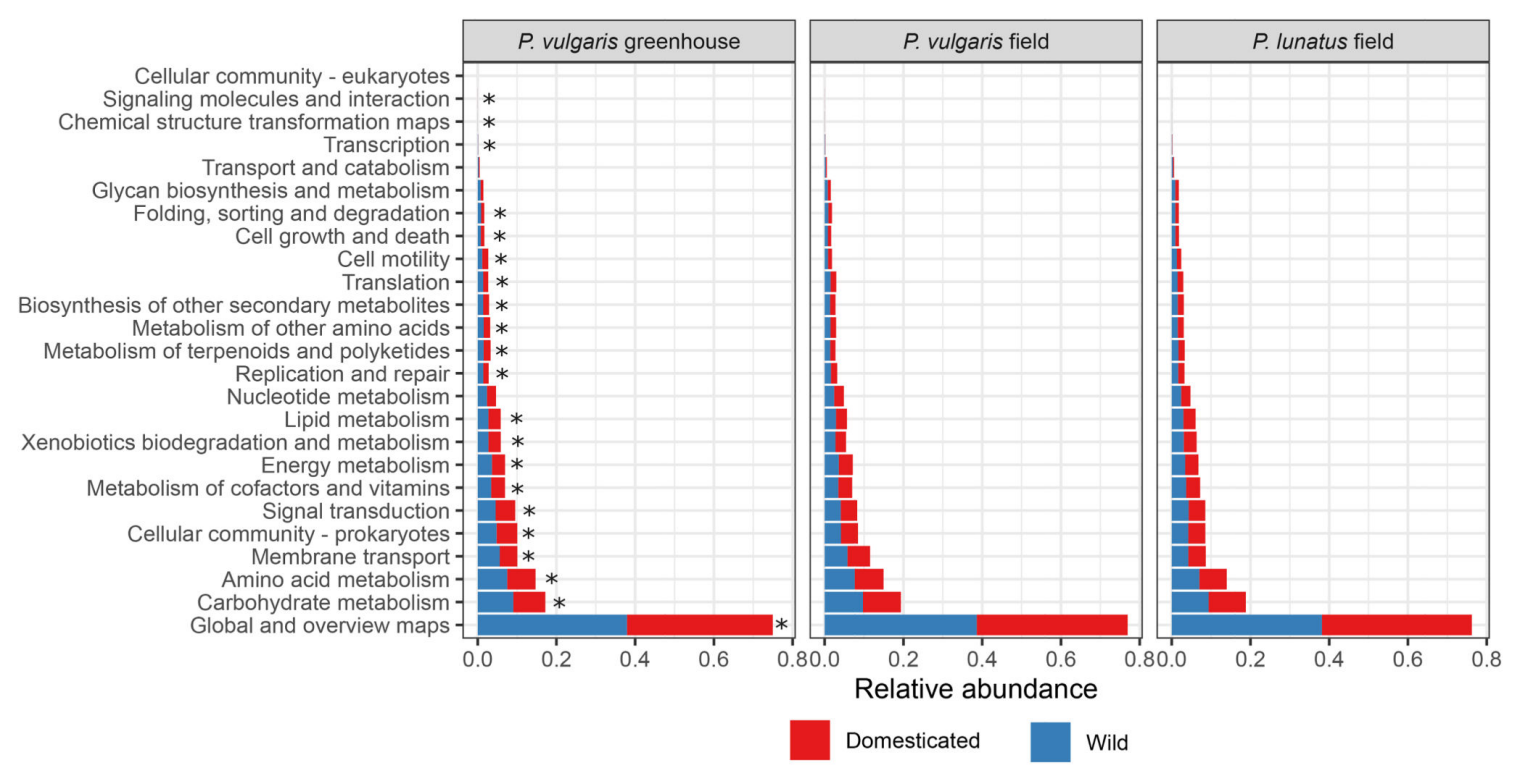
Relative abundance of bacterial metabolic pathways predicted by Tax4Fun2 in wild and domesticated plant samples Metabolic pathways of bacterial communities in wild and domesticated plants predicted by Tax4Fun2. The asterisks indicate significant differences between wild and domesticated plants according to post-hoc analyses (significant level: p < 0.05, with false discovery rate adjustment. PERMANOVA results are reported in the main text).

We predicted functional profiles of the bacterial communities of wild and domesticated plants using Tax4Fun2 and a habitat-specific reference dataset using 456 plant-associated genomes^37^ and found that the bacterial community-level changes between wild and domesticated plants were reflected at a functional taxonomical level (overall difference in the abundance of KEGG Orthology database identifiers) in all three experiments. Permutational analysis of variance (PERMANOVA) was used to assess whether differences in functional profiles between wild and domesticated plants were statistically different (PERMANOVA results for GGPV: df = 1, F = 31.707, p = 0.001***. PERMANOVA results for field-grown *P. vulgaris*: df = 1, F = 1.4623, p = 0.039*. PERMANOVA results for field-grown *P. lunatus*: df = 1, F = 1.6096, p = 0.011*). The results suggest that the effect of plant domestication on microbial communities potentially extends to functional roles. Differences in functional profiles are summarized on KEGG pathways and reported in Figure 5. These included functions associated with cell motility, metabolism, and signaling molecules.

## Discussion

**Figure 6:**
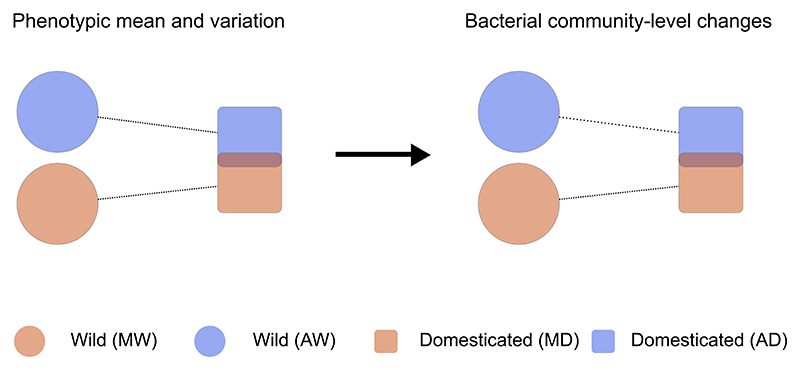
Conceptual model illustrating how the domestication effect on the plant bacterial community is leveraged through domesticated plant phenotypes In this study we found evidence of a statistical relationship between plant traits selected during domestication and bacterial community-level changes. Based on the plant traits measured in this study, domesticated plants have lower phenotypic diversity and similar means compared with their wild counterparts; thus, we can expect that differences in bacterial community composition among domesticated plants are weaker than among wild plants. We report three layers of evidence supporting this hypothesis. First, the overall AIC of the generalized linear models fitted to each SV suggested that using a plant trait as explanatory variable is statistically better than using a qualitative explanatory variable (biological status). Second, the Gaussian copula models confirmed the results of the AIC scores, except for *P. lunatus* where, indeed, phenotypic differences among wild and domesticated plants were weaker than in *P. vulgaris*. Lastly, the random forest classifier provided evidence of a microbial signature resulting from the domestication process, which is independent of the domestication event.

Our results suggest that plant domestication introduced discernible community-level changes in the seed bacteriota of *Phaseolus* spp. that are independent of the domestication event. This appears to be statistically linked to phenotypic changes that occurred during plant domestication (Figure 6). Our results also support the conclusion that because wild plants have higher phenotypic diversity (considering that plant traits are statistically linked to bacterial community-level changes), differences in bacterial community composition among wild plants are likely to be greater than among domesticated plants (Figure 6).

In our study, we focused on measuring quantitative seed traits for which a domestication effect was already reported.^32^ Our results on seed Ca concentration in wild and domesticated seeds of *P. vulgaris* are consistent with previous work that has reported reduced Ca concentrations in domesticated seeds.^32^ However, to the best of our knowledge, no previous studies have investigated differences in seed mineral content between wild and domesticated accessions of *P. lunatus*. We cannot know whether changes in seed mineral content were under direct selection by the domesticator, but genetic determinants of seed Ca concentration in *P. vulgaris* have been identified.^38^ In *P. vulgaris* the majority of Ca in the seed has been reported to be in the seed coat (67%–96% of total seed Ca), and the seed coat is also moderately high in Mg (16%–28% of total seed Mg).^29,39^ High levels of seed coat Ca in *P. vulgaris* have been reported to correlate with increased Ca^2+^-pectic polysaccharide cross-linkage and a ‘hard-to-cook’ phenotype,^40,41^ which suggests the possibility that selection for improved cooking properties has contributed to reduced seed Ca in domesticated plants. However, although Ca has consistently been reported to be more abundant than Mg in the *P. vulgaris* seed coat, the reverse is true for *P. lunatus*.^42^ Statistical evidence for a correlation between domestication status and seed Mg concentrations was less clear cut, but some studies have found positive associations of Mg with crop quality.^43^

Although our goal was not to find a causal relationship between plant phenotypes and microbial members but to find evidence of an independent domestication effect on the plant microbiota via common domesticated plant phenotypes, we highlight that Ca and Mg are important for bacterial spore formation,^44^ affect bacterial membrane and cell wall integrity and antimicrobial resistance, and have been described to significantly influence the composition of gut^45^ and soil microbial communities.^46,47^ Additionally, both cations play significant roles in osmotic stress regulation,^48,49^ which is critical for microbial survival in seeds.^50^

Although we found specific microbial signatures introduced by the domestication process, the environment also strongly influenced plant microbial communities (Figure S3), in agreement with previous studies.^33,51^ Because the plant microbiota is characterized by low inheritance (vertical transmission),^52^ and the environment determines the pool of microorganisms with whom the plant can interact, when we look at the consistency of the domestication effect on seed bacterial communities, we look within experiments and not across. Nonetheless, we found shared OTUs between field and GGPV, but only among wild plants (Figure S3), possibly suggesting lower inheritance or heritability for some members of the microbial communities in domesticated plants.

In this study, we set out to test the hypothesis that plant domestication has influenced the composition of the plant microbiota by examining multiple and independent domestication events within two closely related species. Our results provide evidence that the domestication process resulted in detectable and consistent changes in bacterial community composition and abundance that are independent of the domestication events, which correlate with plant traits that are common across domesticated plants within the same species. This opens up the possibility of better predicting and possibly modifying the composition of the domesticated plant microbiota to improve plant health and productivity.

Here, we focused on seeds, a plant organ that has been directly subject to selection for agriculturally important traits. However, the seed is only one compartment that has been modified through domestication. Further research is needed to holistically assess whether plant domestication effects are plant-compartment dependent and whether changes in seed bacterial communities are primarily linked to seed phenotypes or also linked to phenotypic changes in other compartments through which microorganisms are transmitted to the seed. This would require applying our experimental design to the seed, rhizosphere, and phyllosphere at the same time.

In our analyses, we found the strongest statistical relationships between community composition and seed mineral composition, but it should be emphasized that this is only one of many traits altered through domestication.^10^ Therefore, another important direction for future work will be to examine a wider range of seed traits and their impact on microbiota composition to determine whether changes in plant traits found to be statistically associated with certain microbial features lead to the results predicted by the statistical model.

In previous work, we speculated that changes in plant microbiota interactions arising through domestication could include reduced selection by plants for a beneficial microbiota.^10^ Although in a limited number of cases, it might be feasible to ‘rewild’ the seed microbiome, an equally important approach will be to screen or engineer microbial groups found to consistently associate with domesticated plants and seeds for positive host-to-microbe effects, as bacteria adapted to colonize wild plants may be non-competitive in domesticated plants. The latter approach, although challenging at present due to limitations on the release of GMOs in the environment, might become increasingly feasible with advances in genome editing and biocontainment.

Overall, our study opens up the possibility that the composition and abundance of domesticated plant microbiomes could be consistently predicted within a certain environment and plant species regardless of the domestication event, opening up exciting opportunities to foster the development of new microbial applications in agriculture.

## Star★Methods

### Key Resources Table

**Table:** 

REAGENT or RESOURCE	SOURCE	IDENTIFIER
Biological samples		
*P. vulgaris* and *P. lunatus* accessions, see Data S1	CGIAR-CIAT	N/A
Deposited data		
Code	This study	https://doi.org/10.5281/zenodo.8396606
Processed data	This study	https://doi.org/10.5281/zenodo.8396606
Raw data	This study	https://www.ebi.ac.uk/ena-PRJEB50018
Software and algorithms		
R software	N/A	https://www.r-project.org/

### Resource Availability

#### Lead contact

Further information and requests for resources and reagents should be directed to and will be fulfilled by the lead contact, Gail M.Preston (gail.preston@biology.ox.ac.uk).

#### Materials availability

This study did not generate new unique reagents.

### Experimental Model And Subject Details

#### Plant accession

Plant genotypes were selected based on previous studies of the population genetic structure of *P. vulgaris*^22^ and *P. lunatus* accessions.^18,23^ Only accessions belonging to a specific subpopulation were selected for this study, and admixed accessions were excluded. For the greenhouse experiment, we selected one subpopulation per domestication status and domestication event to have a balanced design (MW, MD, AW, AD). Within each subpopulation, we selected 4 accessions with 7 replicates each. Thus, a total of 112 plants were grown in the greenhouse until maturity, but not all replicates cast seeds (Data S1).

Subsequently, we expanded the analysis to consider 70 different plant genotypes of *P. vulgaris* and 62 of *P. lunatus* encompassing all identified subpopulations, using the same selection criteria, that is that accessions had to belong to a subpopulation.^22,23^ In the case of *P. vulgaris*, all accessions selected for the greenhouse experiment were also included in the second experiment with seeds from plants grown at CGIAR-CIAT. The selected accessions encompass the wide geographical distribution of the genus *Phaseolus* in the Americas. Details of the selected accessions and subpopulation genetic cluster can be found in Data S1. Information on plant phenology was retrieved from CGIAR-CIAT, except for flowering time which was directly recorded for the experiment under controlled conditions. We highlight that in the original greenhouse experiment, we also included *P. lunatus* accessions but a malfunction of the greenhouse heating system caused *P. lunatus* bloom loss.

#### Plant growth conditions and processing

For the greenhouse experiment seeds of *P. vulgaris* were washed in 70% ethanol for 1 minute and rinsed 3 times in sterile water before performing scarification to ease the germination process. After 3 days, germinating seeds were transferred to 3-liter pots containing 40% Norfolk topsoil (https://www.norfolktopsoil.co.uk/product-category/topsoil-and-compost/), 50% vermiculite and 10% sand. Pots were arranged according to complete randomization. Accessions were grown at 20–24°C, with artificial light maintained for 12 h periods within the 24-h cycle until maturity. After 3 weeks, 3 g per pot of fertiliser Floranid Twin Permanent 16-7-15 (Compost Expert, Germany) were added. Drip irrigation was applied to maintain the substrate at field capacity.

At maturity, dry pods were collected and stored at room temperature. Shortly after collection, pods were opened under axenic conditions. Two seeds per pod for a total of 5 pods per plant were used for total DNA extraction, performed 3 months after seed collection. Seeds were crushed in a sterile mortar with liquid nitrogen, under axenic conditions, and 180 mg was used for total DNA extraction.

For experiments carried out using seeds directly coming from CGIAR-CIAT, seeds were washed in 70% ethanol for 1 minute. Ten seeds per sample were processed as described above.

### Method Details

#### Seed chemistry

The chemical properties of the seeds were characterized at Forest Research (UK). Approximately 30 seeds were pooled and crushed in a sterile mortar with liquid nitrogen. Each sample (100 mg) was analyzed for the following elements: calcium, magnesium, potassium, phosphate, zinc, molybdenum, cadmium, aluminum, chrome, copper, nickel and manganese, by using a dual view ICP-OES (Thermo ICap 6500). Results are reported in mg/kg (Data S1). Welch’s t-test was used to assess the differences in mineral concentration means between wild and domesticated seeds because of unequal variances and normality of the distribution.

#### Total DNA extraction

Total DNA was extracted with the Quick-DNA Fecal/Soil Microbe Miniprep Kit (https://zymoresearch.eu/collections/quick-dna-fecal-soil-microbe-kits/products/quick-dna-fecal-soil-microbe-dna-miniprep-kit) (Zymo Research, Irvine, USA) according to the manufacturer’s instructions.

#### Sequencing and Bioinformatics

Investigation of microbial communities was based on paired-end amplicon high-throughput sequencing of the 16S rRNA gene. Amplification was performed with the primers 515F (5’-GTGYCAGCMGCCGCGGTAA-3’) and 806R (5’-GGACTACNVGGGTWTC TAAT-3’).^53,54^ Protein nucleic acid PCR clamps (5 μM) targeting plastidic (pPNA, 5’-GGCTCAACCCTGGACAG-3’) and mitochondrial (mPNA, 5’-GGCAAGTGTTCTTCGGA-3’) DNA (PNA Bio, Newbury Park, CA, USA) were added to samples, as published previously.^55^

All 3 libraries were constructed with the 96 Nextera XT Index Kit (Illumina) following the manufacturer’s instructions with minor modifications. Briefly, the first PCR mixture contents were as follows: Platinum Host-Start PCR Master Mix (2X), 12.5 μl; primers, 1 μl of 10 μM for each; template DNA, 5 μl of 2.5 ng/μl; pPNA and mPNA mix, 5 μl of 25 μM mix; and H_2_O to 25 μl. The PCR conditions were 98°C for 2 min, 33 cycles of 98°C for 15 s, 55°C for 15 s, and 72°C for 17 s, and a final elongation step of 72°C for 2 min. The second amplification was performed according to 96 Nextera XT Index Kit instructions. Library sequencing was performed using the Illumina MiSeq platform with 2x300 pair-end sequencing at the Genomics and Bioinformatics Core Facility, Center for Biomedical Research of la Rioja (Spain). Filter tips were used throughout the library preparation steps alongside controls (water and kit reagents) to detect possible contaminations.

Primers were removed from raw sequencing data using cutadapt.^56^ All further read processing, namely filtering, trimming, merging, and chimeras removal was done in the dada2 package.^57^ Bacterial and archaeal taxonomy was assigned with the naive Bayesian classifier method^58^ implemented in dada2 to the genus and species level using the SILVA reference database v.138. Both NCs (water and kit reagents) contained the same contaminants, 3 SVs belonging to the genera *Escherichia, Paucibacter*, and *Microbacterium*, accounting for > 99% of the reads in the negative controls from both experiments with CGIAR-CIAT grown plants. All SVs found in negative controls (kit reagents) with a relative abundance higher than 0.1% in NC samples were removed from all plant samples. Subsequently, reads belonging to chloroplast and mitochondria were removed. For the first experiment we obtained a mean sequencing depth of 83,860 high-quality bacterial reads per sample while for *P. vulgaris* and *P. lunatus* libraries made from CGIAR-CIAT seeds, we obtained 42,310 and 67,357 bacterial reads per sample respectively. Samples with fewer than 5,000 reads were removed as well as rare SVs (1% prevalence, abundance threshold 20 reads). The SVs table along with metadata information was handled with the R package phyloseq.^59^

### Quantification And Statistical Analyses

#### Statistical analysis and experimental design

We tested whether independent domestication events led to common changes in the seed microbiota composition induced by domesticated plant phenotypes. To answer this question we used two approaches, namely model-based and machine learning applied to 3 independent experiments. The first experiment constituted of accessions grown in the greenhouse (complete randomization) until maturity. We grew representative accessions of domesticated and wild subpopulations for both domestication events (Data S1) to have a balanced design. For the second and third experiments, we expanded the analysis to 70 and 62 plant genotypes for *P. vulgaris* and *P. lunatus*, respectively.

A model-based approach for multivariate data was developed in the R package mvabund.^34^ Model-based multivariate statistics offers several advantages compared to traditional distance-based approaches (e.g. PERMANOVA, ANOSIM, CCA) because it directly accounts for the mean-variance relationship of the data rather than relying on transformation and standardisation.^60^

This approach fits a separate generalized linear model to each SV member of the microbial community, using a common n-dimensional set of explanatory variables. The correlation between taxa is taken into account for the calculation of the p-value using design-based inference. In practice, the statistical significance of the explanatory variable of the fitted models was assessed with ANOVA (likelihood ratio tests) using bootstrap iterations via PIT-trap residual resampling, a method that shows low rates of type I errors.

Since the number of bacterial reads per SV does not reliably reflect bacterial abundance, we introduced an offset for sequencing depth in our model.^61^ We found the best Generalized Linear Model (GLM model, family: negative binomial) based on the sum of the AIC over all variables (seed chemistry and plant phenotypes when appropriate) and accounted for the multivariate feature of the data in the calculation of the p-value with residual resampling. Therefore, our approach was based on model selection using model-based inference and p-value calculation based on design-based inference. Model residuals were checked against fitted values for violation of assumptions prior to model selection. We acknowledge that AIC would require a correlation to be accounted for in model specification to properly account for the multivariate property of the data, but this is not possible in the manyglm function. For this reason, we use AIC as a general guide and further validate the results using Gaussian copula models.^35^

Most of the GLMs (family negative binomial) have been fitted with one explanatory variable and an offset to account for different sequencing depths per sample using:

$$\begin{array}{l} y_{i}\sim NB(\mu_{i},k)\\ E(y_{i})=\mu_{i}\mathrm{var}(y_{i})=\mu_{i}+\frac{\mu_{i}^{2}}{k}\\ \log(y_{i})==\alpha +\beta x+\log(\text{offset}) \end{array}$$

Where k is the dispersion parameter.^62^ The offset has been specified as described in Luo et al.^61^ We automatically included a quadratic term on quantitative explanatory variables if the model with a quadratic term had an overall lower AIC compared to the model without a quadratic term. In the first experiment, we fitted 15 models, 13 models accounted for quantitative variables as explanatory variables (12 related to seed chemistry and 1 to flowering time), and 2 for categorical variables. One categorical variable, named ‘status’ (two levels) indicates whether a plant genotype is domesticated or wild. The categorical variable subpopulation has 4 levels (1 wild subpopulation per domestication event and 1 domesticated subpopulation per domestication event). We repeated a similar analysis for the second and third experiments. In these cases, we included an additional covariate called ‘DS|DE’ which has 4 levels (AD, AW, MD, MW). This is because, for the first experiment, subpopulation corresponded to ‘DS|DE’.

Following the identification of the minimum adequate model based on AIC values, we included as covariates the regeneration site of the accessions and the collection year (CGIAR-CIAT experiments). For models containing 3 covariates (seed phenotype, regeneration site of the accession, collection year) we used 120 bootstrap iterations (120 cores, 1 bootstrap iteration per core). After removing non-significant explanatory variables, we repeated the bootstrapping procedures performing 1,080 iterations (120 cores, 9 bootstrapping per core). We further tested whether the model with the lowest AIC in each experiment explained more co-occurrence patterns than a model accounting for biological status as co-variate. We did so to account for the correlation across taxa, by applying Gaussian copula models.^35^ Briefly, we constructed three models per experiment. An intercept-only model, the model with the lowest AIC (Ca and Mg concentration in the first two and third experiment respectively), and the model with biological status as explanatory variable. We calculated the proportion of explained co-occurrence patterns as described in Warton^63^ (the code is available from GitHub).

A random forest-based classifier with 10 times 5-fold cross-validation was further used to assess whether higher overall accuracy (acc) was reached when classifying wild *vs*. domesticated genotypes based on the seed bacterial community (indicating similar effects of independent domestication events on seed microbiota) than the classification of plant genotypes for each biological status and domestication event (factor with 4 levels; AW, AD, MW, MD). Microbiota data was converted into relative abundances before model fitting, to avoid biases introduced by different sequencing depths per sample.

The random forest-based classifier was built using mlr R package.^64,65^ Good performances of random forest algorithms in microbiome studies have been already reported.^66,67^ Feature selection was performed with the Boruta algorithm with the R package Boruta^68^ with default parameters. Following the identification of the most important features (the most important SVs for the classification task), we randomly subsampled them in combination^69^ and applied random forest with 10 times 5-fold cross-validation resulting in 25,0000 trees. After selecting the model with the highest accuracy, we tuned the hyperparameters (ntree, mtry and nodesize) of the model with the function tuneParams. To demonstrate that the results of the classifier was not strongly influenced by a moderate class imbalance, we also randomly selected 7 samples per group (AD, AW, MW, MD) and repeated the analysis (Figure S1).

Overall, our findings were driven by two very different approaches, namely model-based multivariate statistics and machine learning. Model-based multivariate statistics is a powerful approach to gain insight into factors driving community level changes while machine learning was used to identify the minimum set of microbial members responsible for the classification of samples into wild and domesticated.

#### Bipartite network

We clustered SVs of the 3 libraries into OTUs based on 99% similarities with the R package DECIPHER.^70^ The bipartite network was computed with the script make_bipartite_network.py in qiime^71^ and visualized in Gephi.^72^

#### Phylogenetic tree

For phylogenetic trees in Figure 4, SV sequences were aligned with MAFFT^73^ and the phylogenetic trees constructed with RAxML.^74^ Phylogenetic trees were visualized in iTOL.^75^

#### Functional Profiles

The functional profiles of bacterial communities were predicted using Tax4Fun2.^37^ To increase the accuracy of the predicted functions, as described in Wemheur et al.,^37^ we built an in-house database using 456 plant-associated genomes. The genomes were derived from Midha et al.^76^ and from the Integrated Microbial Genomes and Microbiomes database (https://img.jgi.doe.gov/) downloaded with the following filters: i) Domain=bacteria, ii) host=plant, and iii) high-quality genomes. The functional annotation of these genomes was performed with the Tax4Fun function *assignFunctions*.^37^

PERMANOVA was performed with adonis2^77^ using biological status as an explanatory variable in all three experiments.

## Supplementary Material

Supplementary Materials

## Data Availability

The raw sequencing data are available at https://www.ebi.ac.uk/ena, PRJEB50018. Phyloseq objects, containing SV table, and metadata for each library are deposited at Zenodo. The DOI is listed in the key resources table. All original code has been deposited at Zenodo and is publicly available as of the date of publication. DOIs are listed in the key resources table Any additional information required to reanalyze the data reported in this paper is available from the lead contact upon request.

## Highlights

- Domestication impacts plant microbiota in *Phaseolus* species
- Independent domestication events show similar microbiota changes in *Phaseolus*
- Microbiome shifts in *Phaseolus* link to domestication traits

## In brief

Soldan et al. provide evidence of a domestication effect on the plant microbiota by focusing on multiple and independent domestication events in two *Phaseolus* species. The study links changes in seed microbiome abundance and composition in domesticated plants to the selection of common domesticated plant traits.
