# Supplementary material for: Consistent effects of independent domestication events on the plant microbiota
Source: Curr Biol. Author manuscript; Available in PMC 2025 Aug 1. (PMC7617976; doi:10.1016/j.cub.2023.12.056)
Supplement: Supplementary Materials [file EMS206911-supplement-Supplementary_Materials.pdf]

SUPPLEMENTAL INFORMATION

Supplemental information can be found online at https://doi.org/10.1016/j.cub.2023.12.056.

Current Biology, Volume *34*

Supplemental Information

Consistent effects of independent domestication events on the plant microbiota

Riccardo Soldan, Marco Fusi, Massimiliano Cardinale, Felix Homma, Luis Guillermo Santos, Peter Wenzl, Marcel Bach-Pages, Elena Bitocchi, Maria Isabel Chacon Sanchez, Daniele Daffonchio, and Gail M. Preston

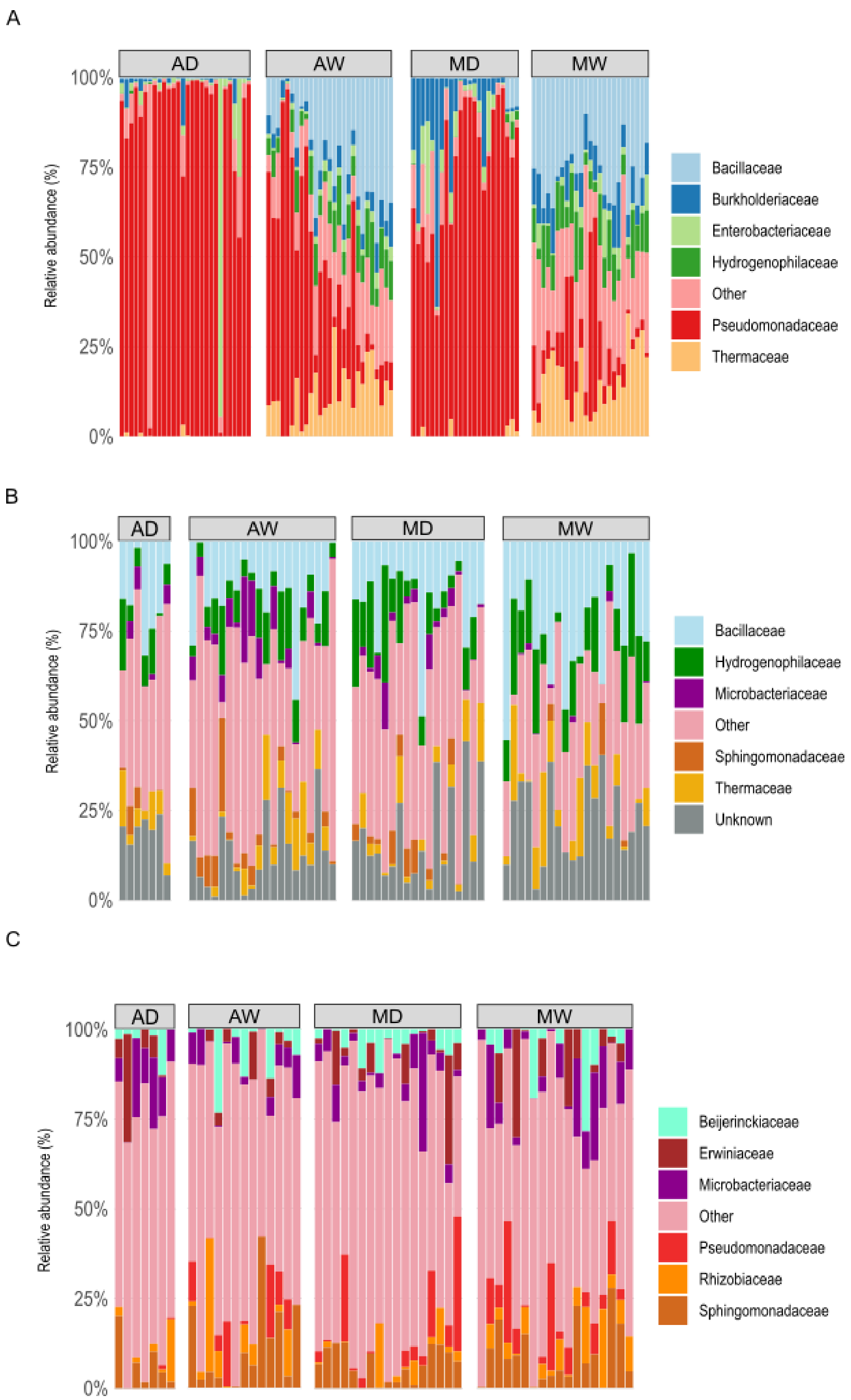

Figure S1. The relative abundance of the most abundant microbial families depends on the environment in which plants are grown. Related to Figures 1 to 3. (A); Greenhouse-grown *P. vulgaris*. **(B);** Field-grown *P. vulgaris*. **(C)**; Field-grown *P. lunatus*. Andean Domesticated (AD), Andean Wild (AW), Mesoamerican Domesticated (MD) and Mesoamerican Wild (MW).

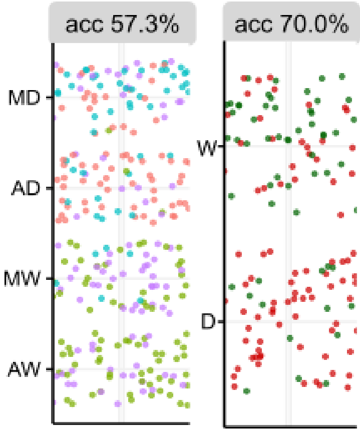

Figure S2. Results of the random forest classifier on a random subselection of samples show that the results are consistent and not influenced by a slight imbalance. Related to Figure 3. Accuracy and confusion matrix of the random forest classifier (10 times 5-fold cross-validation) for classification task domestication status (2 levels; W=wild, D=domesticated with 7 samples for each class, N=28) and domestication status within domestication event (4 levels; AD=Andean Domesticated, AW=Andean Wild, MD=Mesoamerican domesticated and MW=Mesoamerican Wild with 7 samples for each class, N=14) are reported.

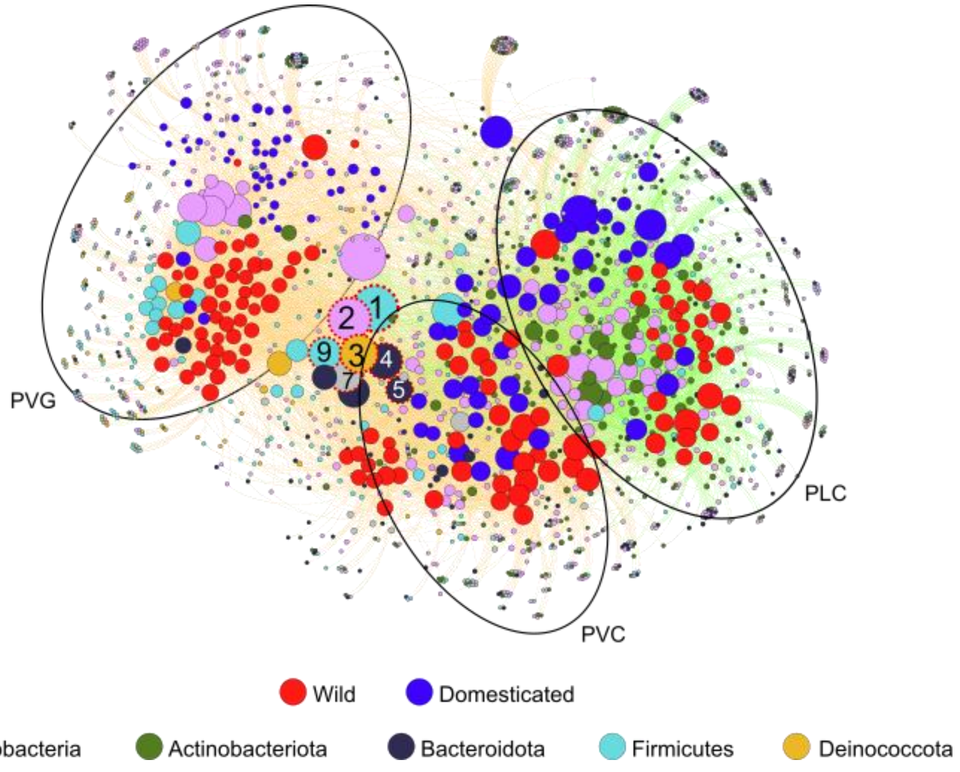

Figure S3. Bipartite network of seed-associated microbial communities. **Related to Figures 1 to 3**. Bipartite network representing sample/OTU interactions. In the network, node size is proportional to the number of degrees (number of connections). Edge color (line connecting different nodes) indicates different plant species (green: *P. lunatus*; orange: *P. vulgaris*). Numbered OTUs indicate shared microbial members between field (PVC experiment) and greenhouse-grown (PVG experiment) wild plants (domesticated plants did not share any OTU). 1 (Family: *Bacillaceae*); 2 (Family: *Hydrogenophylaceae*); 3 (Family: *Thermaceae*); 4 (Order: *Kapabacteriales*); 5 (Class: *Bacteroidia*); 7 (Class: *Blastocatellia*); 9 (Family: *Bacillaceae*). Ellipses highlight the experiment. PVG (*Phaseolus vulgaris* greenhouse); PVC (*Phaseolus vulgaris* CIAT); PLC (*Phaseolus lunatus* CIAT).

**Exp variable**	BIC	% of co-occurrence patterns explained
Ca	101529	31%
Status	104671	26%

Table S1. Results of the Gaussian copula models for the experiment with *P. vulgaris* grown under controlled conditions. Related to Figure 1. Gaussian copula models account for the correlation across taxa and can be used to understand to what extent a certain explanatory variable explains co-occurrence patterns.

**Exp variable**	BIC	% of co-occurrence patterns explained
Ca	79590	19%
Status	80523	10%

Table S2. Results of the Gaussian copula models for the experiment with *P. vulgaris* grown in the field at CGIAR-CIAT. Related to Figure 2. Gaussian copula models account for the correlation across taxa and can be used to understand to what extent a certain explanatory variable explains co-occurrence patterns.

**Covariate**	
Site	LRT=4606, P=0.2
Date	LRT=2076, P=0.13

Table S3. **Deviance and p-value based on Likelihood-Ratio Test (LRT) for the covariates Site and Date for the experiment with *P. vulgaris* grown at CGIAR-CIAT. Related to Figure 2**. Site represents the regeneration site of the accessions and Date, the collection year.

**Exp variable**	BIC	% of correlation among taxa explained
Mg	62228	12%
Status	62795	21%

Table S4. Results of the Gaussian copula models for the experiment with *P. lunatus* grown in the field at CGIAR-CIAT. Related to Figure 3. Gaussian copula models account for the correlation across taxa and can be used to understand to what extent a certain explanatory variable explains co-occurrence patterns.

**Covariate**	
Site	LRT=3276, P=0.09
Date	LRT=1915, P=0.06

Table S5. **Deviance and p-value based on Likelihood-Ratio Test (LRT) for the covariates Site and Date for the experiment with *P. lunatus* grown at CGIAR-CIAT. Related to Figure 3.** Site represents the regeneration site of the accessions and Date, the collection year.

SUPPLEMENTAL REFERENCES

S1. Rodriguez, M., Rau, D., Bitocchi, E., Bellucci, E., Biagetti, E., Carboni, A., Gepts, P., Nanni, L., Papa, R., and Attene, G. (2016). Landscape genetics, adaptive diversity and population structure in *Phaseolus vulgaris*. New Phytol. *209*, 1781–1794. 10.1111/nph.13713.

S2. Chacón-Sánchez, M.I., and Martínez-Castillo, J. (2017). Testing domestication scenarios of Lima Bean (*Phaseolus lunatus* L.) in Mesoamerica: insights from genome-wide genetic markers. Front. Plant Sci. *8*. 10.3389/fpls.2017.01551.
